# Thoughts of an Old Surgeon on the New Mammographic Screening Guidelines

**DOI:** 10.7759/cureus.4439

**Published:** 2019-04-11

**Authors:** Gosta W Iwasiuk

**Affiliations:** 1 Surgery, Community Memorial Hospital, Ventura, USA

**Keywords:** breast cancer awareness, breast cancer screening, statistical confusion, new breast cancer screening guidelines, premature discarding previous guidelines, breast cancer screening guidelines

## Abstract

Several recent reports have concluded that mammography is overrated and overdone. This has brought about a change in the recommendations for screening mammography, from yearly screening starting at age 40, to every other year screening starting at age 50, and no screening after age 74. Furthermore, self-examination is claimed to be next to worthless. Are we treating some cancers that would never grow? That has been known for decades. The problem is we cannot distinguish between the cancers that are lethal and the ones that are harmless because they look the same under the microscope or under any known measure we have to date. Breast cancer remains a formidable enemy! We have made a dent in the death rate since 1989. When you are winning a race, slowing your pace is likely to cost your lead. Before money-saving schemes are implemented, they need to be fully vetted. This has not happened with the new breast cancer screening guidelines.

## Editorial

Several recent recommendations from the American Cancer Society, the US Preventive Services Task Force, and a Harvard study have concluded that mammography is overrated and overdone. The findings indicate that “for every 10,000 women, mammograms probably save 5 lives of women in their 40s, 10 lives of women in their 50s, and 42 lives of women in their 60s.” However, despite the screening, “breast cancer still kills 31 of those screened in their 40s, 62 screened in their 50s, and 88 screened in their 60s. The cumulative risk of a mammogram resulting in a false positive is about 61 percent for a 40 to 50-year-old woman who has annual mammograms for 10 years,” which may “result in needless surgery, chemotherapy, or radiation” [1]. This has brought about a change in the recommendations for screening mammography, from yearly screening starting at age 40, to every other year screening starting at age 50, and not screening after age 74. Furthermore, they say that self-examination is next to worthless.

There are two issues here. One is diagnosis, the second is treatment. First, diagnosis - assuming the studies are accurate, if those lives had not been saved because the diagnosis was not made, the numbers of deaths would look like this: 36 (instead of 31) in the 40s, 72 (instead of 62) in the 50s, and 130 (instead of 88) in the 60s. That is, 16% more deaths in the 40s and 50s group and 48% in the 60s group.

Russian Roulette is a lethal game of chance, originally played by Russian army officers using the Nagant M1895 revolver, the standard issue sidearm of the Russian army. It has seven chambers; only one chamber is loaded with a bullet. The cylinder is spun. The gun is placed on the player’s temple and he pulls the trigger. There is a 14% chance the bullet is in the active chamber and he will die. I recognize that the comparison of Russian Roulette and mammogram statistics are not equivalent, and is meant to be tongue in cheek. But it allows me to quote one of Mark Twain’s most famous one-liners: "There are three kinds of lies, plain lies, damn lies, and statistics” [2]. The statistics on mammography have been gathered since mammography was introduced in 1949. With newer techniques, such as digital imaging, the false positive and false negative rates have dramatically improved. The new guidelines have not had the chance to compare themselves with the decades of past experience. Regarding self-examination, in my nearly half a century of dealing with women’s health issues, a large number of the breast cancers I have seen were discovered by the patient - admittedly not a double-blind controlled study. Self-examination also involves women with the concept that they must take some of the initiative and responsibility for this killer of women. It seems to me that knowledge always trumps ignorance.

In regard to the 61% false positive findings of mammography, that too is a sensational, headline-grabbing, but misleading and biased use of the term false positive. Mammography is just a test that suggests problems. It is not like a hard number that diagnoses cancer. It is similar to a detective finding blood at a suspected crime scene. It does not prove there was a crime or who perpetrated the crime. It does raise the suspicion and that is all. It requires more detective work to investigate what the whole story is. The radiologist even rates the strength of his suspicions in a rank order called Breast Imaging Reporting and Data System (BI-RADS) scoring: 1 and 2 being low risk, 3 possibly cancer, 4 high risk of cancer, 5 being almost certainly cancer, with 6 biopsy-proven cancer. The radiologist also tends to read the films so that his false-negative rate is near zero, i.e. he doesn’t want to miss a cancer. That is, by far, the more important percentage.

The second issue, treatment - if a mammogram detects a suspect cancer, a biopsy is in order. Most biopsies are now done by the radiologist with a thin needle under local anesthesia. This has about as much risk as drinking your morning coffee. It is true that while drinking your coffee, blue ice from an overflying airliner could strike you dead, but the chances are very slim. If the diagnosis of cancer is made, surgery is currently the only known means of curing the disease. Are we treating some cancers that would never grow and cause any harm to the woman? Without question, that is the case. This has been known for decades and is nothing new.

Anecdotal reports from England after National Health was introduced suggested a low recurrence rate of breast cancer after biopsy alone when ductal carcinoma in situ (DCIS) was found. This was supported by rigorous studies done in the US, reviewing 11,760 biopsies done between 1950 to 1968 that found a 28% recurrence rate after biopsy alone when DCIS was diagnosed and a shockingly low recurrence rate of 11% in women over 50 years of age [3].

If no additional treatment was given to these women, a large number of them would undergo an “unnecessary” mastectomy (the standard of care in those years). The only problem was that there was no crystal ball to figure out which woman would go on to develop the invasive breast cancer and which women would be cured. Unfortunately, we, to this date, don’t have that crystal ball either. To say that we are treating women with unnecessary surgery, radiation, and chemotherapy is disingenuous because we do not know which of those cancers are dangerous and which are not. They all look the same under the microscope. To date, no tests can distinguish them! Furthermore, our current treatment options are less invasive and carry less risk than decades ago. Most women now have “lumpectomies” where only the tumor and a small rim of normal tissue is removed.

There are 182,800 new breast cancer cases every year and 41,200 deaths in the US. That is, over one-quarter of women die of the disease. It is the commonest cause of death in women in their 40s, the commonest cause of cancer death in Hispanics, and the second commonest cause of cancer deaths in Caucasian, African-American, and Asian women [4]. Breast cancer remains a very formidable adversary! Breast cancer death rates have decreased since 1989, largely in women under 50 [5]. This would be from early detection and better treatment and not from a decrease in estrogen use, which could account for the over 50 population! We have made a dent. When you are winning a race, slowing your pace down is likely to cost you your lead. Before money-saving schemes are implemented, they need to be fully vetted. This has not yet happened with the new breast cancer screening guidelines.
